# Insulin-like growth factor-1 genotypes and haplotypes influence the survival of prostate cancer patients with bone metastasis at initial diagnosis

**DOI:** 10.1186/1471-2407-13-150

**Published:** 2013-03-25

**Authors:** Norihiko Tsuchiya, Shintaro Narita, Takamitsu Inoue, Mitsuru Saito, Kazuyuki Numakura, Mingguo Huang, Shingo Hatakeyama, Shigeru Satoh, Seiichi Saito, Chikara Ohyama, Yoichi Arai, Osamu Ogawa, Tomonori Habuchi

**Affiliations:** 1 Department of Urology, Akita University Graduate School of Medicine, 1-1-1 Hondo, Akita, 010-8543, Japan; 2 Department of Urology, Hirosaki University Graduate School of Medicine, 5 Zaifu-cho, Hirosaki, Aomori, 036-8562, Japan; 3 Division of Urology, Department of Organ-oriented Medicine, University of the Ryukyu, 207 Uehara, Nishihara, Okinawa, 903-0215, Japan; 4 Department of Urology, Tohoku University Graduate School of Medicine, 2-1 Seiryo-machi, Aoba-ku, Sendai, 980-8575, Japan; 5 Department of Urology, Graduate School of Medicine, Kyoto University, 54 Syogoin-kawaramachi, Sakyo-ku, Kyoto, 606-8507, Japan

**Keywords:** Prostate cancer, Bone metastasis, Survival, Polymorphism, Insulin-like growth factor-1

## Abstract

**Background:**

The insulin-like growth factor-1 (IGF-1) plays an important role in growth of prostate cancer (PCa) cells and facilitating the development and progression of PCa. This study aimed to evaluate the association of polymorphisms in three linkage disequilibrium (LD) blocks of the IGF-1 on the survival of metastatic PCa patients.

**Methods:**

A total of 215 patients with bone metastases at initial presentation were included in this study. The cytosine-adenine (*CA*) repeat polymorphism and rs12423791 were selected as representative polymorphisms in the LD blocks 1 and 2, respectively. Haplotype in the LD block 3 was analyzed using two tag single nucleotide polymorphisms (SNPs), rs6220 and rs7136446. Cancer-specific survival rate was estimated from the Kaplan-Meier curve, and the survival data were compared using the log-rank test.

**Results:**

Cancer-specific survival was significantly associated with the *CA* repeat polymorphism, rs12423791, and rs6220 (*P =* 0.013, 0.014, and 0.014, respectively). Although rs7136446 had no significant association with survival, the haplotype in the LD block 3 was significantly associated with cancer-specific survival (*P =* 0.0003). When the sum of the risk genetic factors in each LD block (19-repeat allele, *C* allele of rs12423791, or *C-T* haplotype) was considered, patients with all the risk factors had significantly shorter cancer specific-survival than those with 0–2 risk factors (*P =* 0.0003).

**Conclusions:**

Polymorphisms in the *IGF-1*, especially a haplotype in the LD block 3, are assumed to be genetic markers predicting the outcome of metastatic PCa.

## Background

Prostate cancer is typically a type of slow-growing cancer and generally well controlled by endocrine therapies even if distant metastases are present. However, those patients with distant metastases exhibit disease progression within 12 to 18 months on average and gradually manifest resistant to endocrine therapies thereafter [1]. Because several new promising agents are available or being tested for treatment of castration-resistant prostate cancer [2-4], it has been of importance to identify pretreatment prognostic factors in metastatic prostate cancer for adjusting treatment intensity in each patient. Clinical and laboratory factors such as extent of disease (EOD) score [5], serum alkaline phosphate (ALP) [6], hemoglobin (HGB) [7], and prostate specific antigen (PSA) [8] have been used as prognostic markers for those patients since 20 years ago. Recent studies suggest that patients’ intrinsic genetic factors or an interaction with environmental factors may have an impact on progression or survival in advanced prostate cancer patients [9,10]. Our previous study demonstrated that the *insulin-like growth factor-1* (*IGF-1*) and the *cytochrome P450 aromatase* (*CYP19*) polymorphisms were significantly associated with the cancer -specific survival of metastatic prostate cancer [10]. However, to date, investigations of genetic polymorphisms associated with cancer progression or survival have just begun and only a few reports are available in regard to a prostate cancer [11,12]. Evaluating outcomes using genetic makers combined with conventional prognostic markers is expected to lead to more accurate prediction of response to treatments or survival.

IGF-1 is involved in embryonic growth, homeostasis, and various diseases by regulating cell differentiation, proliferation, migration, and apoptosis. In the prostate, it plays an important role in the growth of both normal and cancer cells and facilitates the development and progression of prostate cancer [13,14]. Recent meta-analysis revealed that men with higher circulating IGF-1 levels had an increased risk of prostate cancer compared with men with lower IGF-1 levels [15] and the levels of circulating IGF-1 had a heritable component [16]. The same positive association was observed between circulating IGF-1 level and a risk for breast and colorectal cancer [17-21]. Meanwhile, a cytosine-adenine (*CA*) repeat polymorphism has been known to be located in the promoter region of the *IGF-1* gene [22] and many studies investigated the influence of the polymorphism on the circulating IGF-1 level and risk for certain types of cancer [23]. Recent genome research revealed a number of single nucleotide polymorphism (SNP) throughout the *IGF-1* region and haplotype analyses demonstrated that those SNPs were divided into three to four blocks in which SNPs for each block are in linkage disequilibrium (LD) each other [24,25]. Especially a haplotype in the LD block 3 located in a downstream of the *CA* repeat has been suggested as a novel genetic variation associated with circulating IGF-1 level or cancer risk [25,26].

In this retrospective study, we aimed to evaluate the association of four polymorphisms in three LD blocks of the *IGF-1* on the survival of prostate cancer patients with bone metastasis at initial diagnosis.

## Methods

### Patients

From July 1980 to September 2008, 215 native Japanese patients of prostate cancer with bone metastasis at initial presentation in Akita University Hospital and its related community hospitals, Kyoto University and Tohoku University Hospitals were enrolled in this study. Pathological diagnosis was made by prostate needle biopsy specimens and metastasis was identified by X-rays, CT scans, or bone scintigraphy. All the patients had no previous treatments at presentation and underwent surgical castration or luteinizing hormone-releasing hormone (LH-RH) analogues with or without antiandrogens as primary endocrine therapy. When the treatment failure was observed, optional therapies, including other antiandrogens, estrogens, steroids, chemotherapeutic agents, palliative radiation, or a combination of these was added or replaced.

Pathological grading of needle biopsy specimens was performed according to Gleason grading system by local pathologists with no designated primary pathologist. In 10 patients, the final pathological grade was not determined because no grade information was described in the final report or a different grading system was applied by the local pathologists. Pretreatment HGB, ALP, lactate dehydrogenase (LDH), and PSA levels before the initial treatment of prostate cancer were obtained from medical charts. An independent end-point reviewer in each institution determined the cause of death on the basis of standardized extractions from the patients’ medical files without providing genotype data of each patient.

Written informed consent was obtained from all the patients enrolled in this study for the use of their DNA and clinical information. This study was approved by the Institutional Review Board (the Ethical Committee) in each institution.

### Genotyping analysis

**Figure 1:**
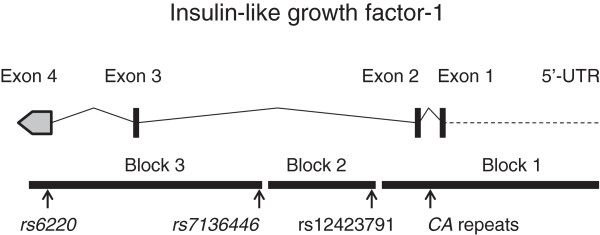
**Genomic structure of the *IGF1 *gene.** The map shows the structure of the *IGF-1* gene and its linkage disequilibrium (LD) blocks. The locations of four polymorphisms analyzed in this study are indicated in the figure.

DNA was extracted from a peripheral blood sample of each patient using a QIAamp Blood Kit (QIAGEN, Hilden, Germany) or standard phenol-chloroform. We divided the *IGF-1* gene into three LD blocks according to a previous report by Johansson et al. (Figure 1) [25]. The representative polymorphisms in each LD block were chosen with reference to literatures as the genes previously described to be associated with the circulating IGF-1 level or the increased risk of prostate cancer [27] and SNP database of international HapMap project (http://hapmap.ncbi.nlm.nih.gov/) [28]. The *CA* repeat polymorphism in the promoter region and rs12423791 [GenBank] were selected from representative polymorphisms in the LD block 1 and 2, respectively. rs6220 [GenBank] and rs7136446 [GenBank] were selected as tag SNPs for a haplotype analysis in the LD block3. Genotypes of the *CA* repeat polymorphism were determined by an automated sequencer (ABI PRISM 310 Genetic Analyzer) with GENESCAN software (Applied Biosystems, Foster City, CA) as described previously [17]. Other three SNPs were genotyped using polymerase chain reaction (PCR)-restriction fragment length polymorphism (RFLP) method. The sequence of forward and reverse primers used for genotyping of the SNPs were as follows; 5^′^-GCTGCTTCTTCCAATGAGAG-3^′^ and 5^′^-GAAAAGCATGTTGCTGCCTC-3^′^ for rs12423791 (123 bp), and TGCCTAGAAAAGAAGGAATC-3^′^ and 5^′^-TGACTCTTCTATGCAGTTAC-3^′^ for rs6220 (105 bp), and 5^′^-CTTCTTGCAGAACTAAGCTCAAGTC-3^′^ and 5^′^-GCCTATTCATTTTCACATACTACCC-3^′^ for rs7136446 (126 bp). Each PCR product was digested with DdeI, MspI, and MnlI, respectively, overnight at 37°C, and electrophoresed on 3.0% agarose gels to determine the genotype. Several samples were directly sequenced using Dye Terminator Sequencing Kit version 1.0 (PE Applied Biosystems) on an ABI prism 310 auto-sequencer to confirm the results of PCR-RFLP for each polymorphism.

### Statistical analysis

The endpoint of this study was prostate cancer-specific survival. The survival time was calculated from the date of prostate cancer diagnosis to the date of death or the last contact with patients. To compare the survival, patients were dichotomized by the median value of age and PSA, by normal limits in HGB, ALP, LDH, by the tumor grade system (Gleason score). Haplotypes of the LD block 3 defined two SNPs, rs6220 and rs7136446, were inferred using expectation-maximization algorithm in SNPAlyze software ver.7 (Dynacom Co. Ltd., Chiba, Japan). Differences in survival between groups were analyzed using the logrank test. For the *CA* repeat polymorphism, the number of repeats was dichotomized as having or not having the 19-repeat allele. Each SNP or haplotype was evaluated using dominant, recessive, and addictive model, and the most statistically significant model was selected. The *IGF-1* polymorphisms and clinicopathological prognostic factors were assessed by the Cox proportional hazard regression models. Age, Gleason score, PSA, HGB, ALP, LDH, and the LD block 3 haplotype were employed as a variable set in a full-variable model of multivariate analysis. Of the variables, Gleason score, HGB, ALP, and the LD block 3 haplotype were selected in a reduced variable model. Cancer-specific survival was estimated using the Kaplan–Meier method. All the statistical analyses were performed using SPSS software version 19.0 (IBM Japan Ltd., Tokyo, Japan) and two-sided *P* values of less than 0.05 were considered to indicate statistical significance.

## Results

### Clinicopathological background of patients

The mean age (± SD) of the 215 patients was 70.2 ± 8.4 years (range, 45–89; median, 72 years). The mean follow-up period was 46.4 ± 36.1 months (range, 1–209; median, 37 months). Of 215 patients, bone metastasis alone, additional lymph nodes metastasis, and other visceral metastasis were seen at initial diagnosis in 106 (49.3%), 98 (45.6%), and 15 (7.0%), respectively. The distribution of Gleason score of biopsy specimen was < 7 in 15 patients (7.0%), 7–8 in 81 (37.7%), 9–10 in 109 (50.7%), and unknown in 10 (4.6%). Pretreatment PSA, HGB, ALP, and LDH levels are shown in Table 1. All the patients were received an endocrine therapy as an initial treatment, surgical castration alone in 29 (13.5%), LH-RH analogue alone in 53 (24.6%), combined androgen blockade in 131 (60.9%). Among 188 patients with available data, 91patinets (48.4%) achieved a PSA nadir less than 1 ng/ml, while 97 patients (51.6%) did not reach the level after initial endocrine therapies.

**Table 1:** Patients’ clinical characteristics

	**Mean ± SD (median)**	**Range**
Age (years)	70.2 ± 8.4 (72)	45 – 89
PSA (ng/mL)	1,029 ± 1,896 (260)	2.4 – 12,490
HGB (g/dL)	13.1 ± 1.9 (13.3)	7.4 – 17.4
ALP (IU/L)	615 ± 908 (291)	50 – 5,870
LDH (IU/L)	295 ± 180 (222)	97 – 1,273
Follow-up (months)	46.4 ± 36.1 (37)	1 – 209
	**N (%)**	
Metastases		
Bone only	106 (49.3)	
Lymph nodes	98 (45.6)	
Other organs	15 (7.0)	
Gleason score		
< 7	15 (7.0)	
7 – 8	81 (37.7)	
9 – 10	109 (50.7)	
Unknown	10 (4.6)	

### Genotyping analysis

The repeat number of the *CA* repeat polymorphism ranged from 13 to 20, and 20 genotypes were observed. The distributions of the genotypes of the *CA* repeat polymorphisms, rs12423791, rs6220, and rs7136446 were shown in Table 2. Estimated haplotype frequencies of the LD block 3 (rs6220 - rs7136446) were 55.2% (*T-T*), 26.7% (*C-T*), 16.8% (*C-C*), and 1.3% (*T-C*). Thirty-seven patients with heterozygous genotype of both rs6220 and rs7136446 were estimated as having the *T-T* and *C-C* haplotypes because estimated haplotype frequency of the *T-C* haplotype was quite low. Haplotypes in all other patients were uniquely determined.

**Table 2:** Genotype distributions of four polymorphisms analyzed in this study

**Genotype**	**N (%)**
*CA* repeat	
13/15	1 (0.5)
14/17	1 (0.5)
15/16	4 (1.9)
15/17	11 (5.1)
15/18	3 (1.4)
15/19	12 (5.6)
15/20	1 (0.5)
16/16	2 (0.9)
16/17	25 (11.6)
16/18	7 (3.3)
16/19	17 (7.9)
17/17	16 (7.4)
17/18	18 (8.4)
17/19	48 (22.3)
17/20	1 (0.5)
18/18	9 (4.2)
18/19	16 (7.4)
18/20	1 (0.5)
19/19	15 (0.5)
19/20	7 (7.0)
rs12423791	
*GG*	140 (65.1)
*GC*	75 (34.9)
rs6220	
*TT*	71 (33.0)
*TC*	102 (47.5)
*CC*	42 (19.5)
rs7136446	
*TT*	146 (67.9)
*TC*	60 (27.9)
*CC*	9 (4.2)

### Survival analysis

**Figure 2:**
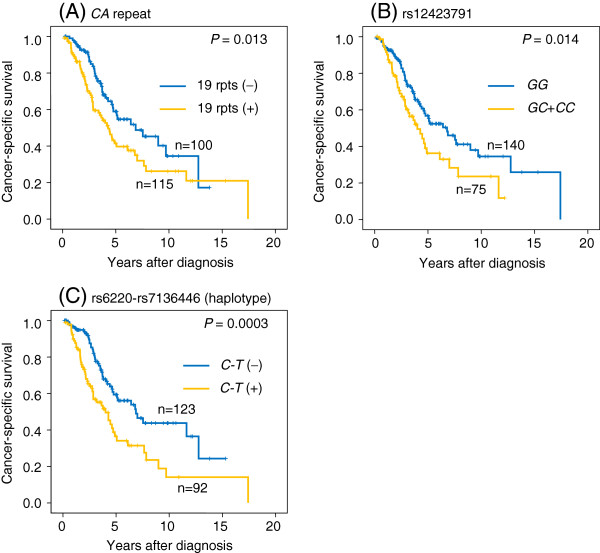
**Cancer-specific survival of patients classified based on the risk allele, genotype or haplotype.** The survival was significantly worse in patients with 19 *CA* repeat allele in the LD block1 than those without 19 *CA* repeat allele (*P* = 0.013) (**A**). Patients with *GC* or *CC* genotype of the rs12423791 in the LD block 2 had significantly worse survival than those with *GG* genotype (*P* = 0.014) (**B**). As regards to the LD block 3 haplotype, patients with *C*-*T* haplotype had significantly worse survival than those without *C*-*T* haplotype (*P* = 0.0003) (**C**).

**Figure 3:**
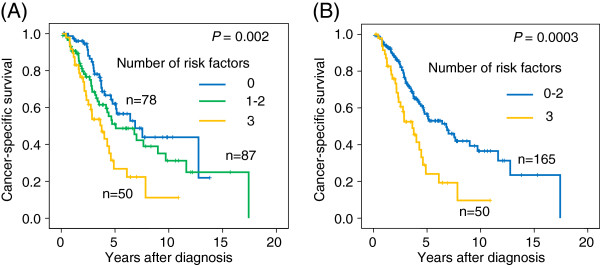
**Cancer-specific survival of patients classified based on the sum of genetic risk factors.** The genetic risk factors were defined as presence of 19 repeat allele of the *CA* repeat polymorphism, *C* allele of the rs12423791, and *C*-*T* haplotype of the LD block 3. Each patient was assigned to one of 3 groups (0, 1–2, or 3 risk factors) (**A**) or one of 2 groups (0–2 or 3 risk factors) (**B**). The cancer-specific survivals significantly differed in both of the classification (*P* = 0.002 and 0.0003).

Kaplan-Meier curves demonstrated that patients with 19-repeat allele, *C* allele of rs12423791, or *C* allele of rs6220 had a significantly worse survival (*P =* 0.013, 0.014, or 0.014, respectively). Whereas, rs7136446 was not associated with patients’ survival (*P =* 0.371). Patients with at least one *C-T* haplotype showed significantly worse survival compared with those who had no *C-T* haplotype (*P =* 0.0003) (Figure 2). When the number of the genetic risk factors (19-repeat allele, *C* allele of rs12423791, or *C-T* haplotype) was considered, cancer-specific survival significantly shortened with increased the number of genetic risk factors (*P =* 0.002), and patients with all the genetic risk factors had significantly shorter survival than those with 0–2 risk factors (*P =* 0.0003) (Figure 3).

An univariate Cox proportional hazard regression analysis showed that cancer-specific survival was significantly lower in patients with Gleason score of 9 or higher (HR: 1.759, 95% CI: 1.151-2.687, *P =* 0.009), HGB less than 11.5 g/dl (HR: 2.251, 95% CI: 1.261-4.019, *P =* 0.006), ALP of 350 IU/ml or higher (HR: 2.836, 95% CI: 1.756-4.578, *P =* 0.00002), or LDH of 500 IU/ml or higher (HR: 2.638, 95% CI: 1.442-4.829, *P =* 0.002) (Table 3). Meanwhile, neither dichotomized age nor PSA was associated with cancer-specific survival. In a multivariate analysis including all the clinicopathological variables and haplotype of the LD block 3 as a representative genetic variable, higher Gleason score (HR: 1.766, 95% CI: 1.052-2.966, *P =* 0.031), higher ALP (HR: 2.598, 95% CI: 1.483-4.551, *P =*0.0008), and the *C-T* haplotype (HR: 2.619, 95% CI: 1.559-4.399, *P =* 0.0003) were independent factors predicting cancer-specific survival. HGB and LDH showed borderline significance (*P =* 0.061 and 0.059, respectively). In a reduced variable model, higher ALP (HR: 2.819, 95% CI: 1.695-4.689, *P =* 0.00007) and *C*-*T* haplotype (HR: 2.626, 95% CI: 1.603-4.305, *P =* 0.0001) were stronger independent predictors for the survival, followed by HGB (HR: 2.082, 95% CI 1.113-3.897, *P =* 0.022) and Gleason score (HR: 1.709, 95% CI: 1.054-2.771, *P =* 0.030) (Table 3).

**Table 3:** Univariate and multivariate analysis of clinicopathological and genetic factors predicting cancer-specific survival

***Univariate analysis***				
	**Category**	**HR**^**1**^	**95% CI**^**2**^	***P***
Clinical and pathological factors				
Age (yrs)	≥ 72 vs. < 72	1.183	0.790 - 1.772	0.415
Gleason score	≥ 9 vs. < 9	1.759	1.151 – 2.687	0.009
PSA (ng/mL)	≥ 260 vs. < 260	1.510	0.992 – 2.299	0.054
HGB (g/dL)	< 11.5 vs. ≥ 11.5	2.251	1.261 – 4.019	0.006
ALP (IU/L)	≥ 350 vs. < 350	2.836	1.756 – 4.578	0.00002
LDH (IU/L)	≥ 500 vs. < 500	2.638	1.442 – 4.829	0.002
Genetic factors				
LD block 1 (*CA* repeat)	19 rpts (+) vs. (-)	1.671	1.109 – 2.518	0.014
LD block 2 (rs12423791)	*GC* + *CC* vs. *GG*	1.658	1.102 – 2.495	0.015
LD block 3 (haplotype)	*C-T* (+) vs. *C-T* (-)	2.054	1.373 – 3.075	0.0005
Number of risk factors	0 vs. 1–2 vs. 3	1.578	1.208 – 2.060	0.0008
	0–2 vs. 3	2.202	1.414 – 3.430	0.0005
***Multivariate analysis***				
Full model				
Age (yrs)	≥ 72 vs. < 72	0.919	0.554 – 1.526	0.745
Gleason score	≥ 9 vs. < 9	1.766	1.052 – 2.966	0.031
PSA (ng/mL)	≥ 265 vs. < 265	0.932	0.496 – 1.749	0.826
HGB (g/dL)	< 11.5 vs. ≥ 11.5	2.012	0.968 – 4.180	0.061
ALP (IU/L)	≥ 350 vs. < 350	2.598	1.483 – 4.551	0.0008
LDH (IU/L)	≥ 500 vs. < 500	1.836	0.977 – 3.448	0.059
LD Block 3 (haplotype)	*C-T* (+) vs. *C-T* (-)	2.619	1.559 – 4.399	0.0003
Reduced model				
Gleason score	≥ 9 vs. < 9	1.709	1.054 – 2.771	0.030
HGB (g/dL)	< 11.5 vs. ≥ 11.5	2.082	1.113 – 3.897	0.022
ALP (IU/L)	≥ 350 vs. < 350	2.819	1.695 – 4.689	0.00007
LD Block 3 (haplotype)	*C-T* (+) vs. *C-T* (-)	2.626	1.603 – 4.305	0.0001

^1^HR, hazard ratio; ^2^95% CI, 95% confidence interval.

**Figure 4:**
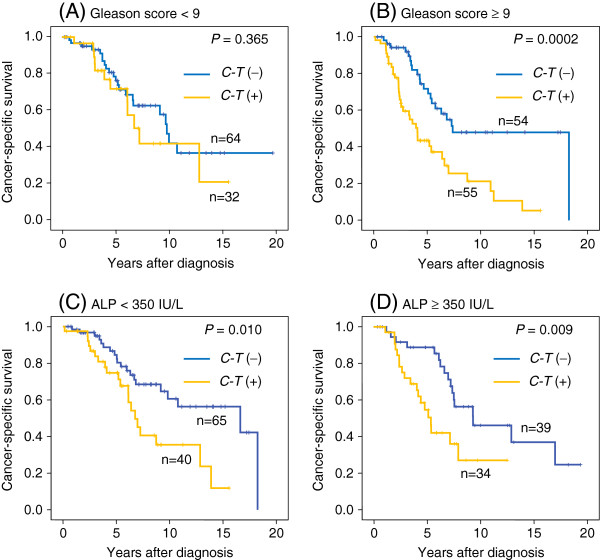
**Cancer-specific survival of patients classified based on haplotype in the LD block 3 in subgroups dichotomized by Gleason score or pretreatment ALP level.** Patients with *C*-*T* haplotype showed significantly worse survival than those having no *C*-*T* haplotype (*P* = 0.0002) in patients with Gleason score of 9–10 (**A**), while there was no significant difference (*P* = 0.365) in patients with Gleason score less than 9 (**B**). In a subgroup analysis by pretreatment ALP level, patients with *C*-*T* haplotype showed significantly shorter survival than those with no *C*-*T* haplotype in either subgroup of patients with lower (< 350 IU/L) or higher (≥ 350 IU/L) ALP (*P* = 0.010 or 0.009, respectively) (**C** and **D**).

Because the Gleason score and the pretreatment ALP level were shown to be significant prognostic factors along with the LD Block 3 haplotype by multivariate analysis (Table 3), we performed subgroup analyses according to the dichotomized Gleason score or the dichotomized pretreatment ALP level to compare survivals by presence or absence of *C-T* haplotype. Among patients with Gleason score of 9–10 (n = 108), those with C-T haplotype showed significantly worse survival than those having no *C-T* haplotype (*P =* 0.0002), while there was no significant difference (*P =* 0.365) in patients with Gleason score less than 9 (n = 96). Regarding ALP, patients with *C-T* haplotype showed significantly shorter survival than those with no *C-T* haplotype in either subgroup (ALP higher or lower than 350 IU/ml) (*P =* 0.010 or 0.009, respectively) (Figure 4).

## Discussion

The association of the *CA* repeat polymorphism in the promoter region of the *IGF-1* with circulating IGF-1 levels and a risk of breast, prostate, and colorectal cancers have been extensively evaluated [23,29]. In those previous studies, the *CA* repeat polymorphisms were generally categorized as having a 19-repeat allele or not having the allele. To date, however, the results were inconsistent in terms of whether 19-repeat allele increases IGF-1 levels or cancer risks. A recent large-scale study of 6,400 healthy subjects indicated that other polymorphisms downstream of the *CA* repeat polymorphism may affect IGF-1 circulation levels [30]. In prostate cancer patients, Johansson et al. demonstrated that heterozygous haplotype of *T-C-C* (rs6220-rs7136446-rs2033178 [GenBank]) in the 3^′^ region of the *IGF-1*, which was a risk haplotype of prostate cancer risk in their previous study, was significantly associated with higher circulating levels of IGF-1 [27]. In the study, however, another cohort did not show the significant difference in circulating IGF-1 levels and rather patients with rs6220 *CC* genotype showed significantly higher circulating IGF-1 level in a separate SNP analysis of each SNP [27]. Other study also showed significantly increased circulating IGF-1 levels in females with rs6220 *CC* genotype [31]. Since all the patients in our series had the *CC* genotype at rs2033178 (data not shown), the *T-C-C* haplotype is referred to as the *T-C* haplotype in our study. Although the *T-C* (*T-C-C*) haplotype was not separately analyzed in a survival analysis due to the rare haplotype with only 1.3%, our result showed that patients with the *C-T* haplotype had significantly worse survival and appeared to be in line with previous studies investigating the association between circulating IGF-1 levels and the *IGF-1* polymorphisms.

Multiple interpretations are possible regarding the role of the polymorphisms in altering the circulating IGF-1 levels. A previous study demonstrated in other genes that the *CA* repeat in the promoter region acted as a negative control element [32], suggesting a possibility that a *CA* repeat length directly affects the transcriptional activity of the *IGF-1*. This hypothesis is partially supported by a study conducted by Missmer et al. [33]. They showed a trend of decreasing IGF-1 level with increasing the *CA* repeat length genotype, although this was not statistically significant [33]. Another explanation is that *CA* repeats do not directly affect the transcriptional activity but other SNPs being in linkage disequilibrium exert functional effect on a transcriptional activity. Recent studies demonstrated that SNPs or haplotypes in other regions in the *IGF-1*, especially downstream of the *CA* repeats, were associated with circulating IGF-1 levels or cancer susceptibility [24,30]. Chen et al. reported a possible association of a haplotype combined of SNPs and the *CA* repeat length with circulation IGF-1 levels. In the study, the combined haplotype was correlated with circulating IGF-1 levels and neither SNPs nor the *CA* repeat alone was associated with the IGF-1 levels [34]. Nevertheless, in vitro studies are needed to determine the functional implication of those genetic polymorphisms on the alteration of IGF-1 expression.

Several mechanisms of IGF-1 affecting the prognosis of metastatic prostate cancer are envisioned. First, IGF-1 is known to act as an important growth factor regulating proliferation and apoptosis of cancer cells and has a role in an acquisition of resistance to endocrine therapies [35-37]. Secondary, IGF-1, which is also produced by bone cells, down-regulates osteoprotegerin (OPG) and up-regulated receptor activator of NF-κB ligand (RANKL) [38]. Results of the present study suggest that the polymorphisms are associated with an aggressive phenotype and resistance to endocrine therapy and facilitate the progression of prostate cancer cells especially in bone metastasis.

The present study has several limitations. First, the present study has a possible bias of patient selection, which is a drawback of retrospective study design. Although the majority of the patients are incident cases, some patients who had rapid progression and very short-term survival may have not been enrolled into the study. Second, treatment strategy was not regulated in this retrospective study. Because of the long recruiting period, various treatments except for endocrine therapies including docetaxel were administered only in recent cases. A prospective study with a large cohort is mandatory to validate the results. Thirdly, we examined only 4 polymorphism loci and other SNPs may have a stronger association with the survival than those evaluated in the present study. A study using precise SNP panel may lead to identify SNPs truly responsible for the survival and the function of the SNPs should be supported by biological investigations.

## Conclusions

Polymorphisms of the *IGF-1*, especially *C-T* haplotype in the LD block 3 were associated with worse survival of prostate cancer patients with bone metastasis at initial diagnosis. The genomic variations in the *IGF-1* combined with conventional clinicopathological prognostic markers, along with conventional clinical markers, appeared to be useful for predicting the outcome of metastatic prostate cancer.

## Abbreviations

EOD: Extent of disease; ALP: Alkaline phosphate; HGB: Hemoglobin; PSA: Prostate specific antigen; IGF-1: Insulin-like growth factor-1; CYP19: Cytochrome P450 aromatase; SNP: Single nucleotide polymorphism; LH-RH: Luteinizing hormone-releasing hormone; LDH: Lactate dehydrogenase; PCR: Polymerase chain reaction; RFLP: Restriction fragment length polymorphism; RANKL: Receptor activator of NF-κB ligand

## Competing interests

The authors declare that they have no competing interests.

## Authors’ contributions

NT and TH were involved in the conception and design of the study. SN, TI, and MH performed laboratory work. MS, KN, SS, SH, and OO were involved in the provision of study material and patients’ clinical data. NT and TH drafted the manuscript. SS, CO, YA, OO supported the manuscript writing. All authors have read and approved the final manuscript.

## Pre-publication history

The pre-publication history for this paper can be accessed here:

http://www.biomedcentral.com/1471-2407/13/150/prepub
